# Measurement of hepatitis B virus DNA in fresh versus processed dentin from chronically infected patients

**DOI:** 10.1186/s12967-018-1719-9

**Published:** 2018-12-12

**Authors:** Inwoong Um, Sungweon Choi, Youngkyun Kim, Kangmi Pang, Jongho Lee, Minsun Lee, Bongju Kim

**Affiliations:** 1 R&D Institute, Korea Tooth Bank, 622 Eonju-ro, Gangnam-gu, Seoul, 06101 South Korea; 2 Oral Oncology Clinic Research Institute and Hospital National Cancer Center, 323 Ilsan-ro, Ilsandong-gu, Goyang-si, Gyeonggi-do 10408 South Korea; 3 Department of Oral and Maxillofacial Surgery, Seoul National University Bundang Hospital, 82 Gumi-ro 173beon-gil, Bundang-gu, Seongnam-si, Gyeonggi-do 13620 South Korea; 4 Department of Oral and Maxillofacial Surgery, Seoul National University Gwanak Dental Hospital, 1 Gwanak-ro, Gwanak-gu, Seoul, 08826 South Korea; 5 Clinical Trial Center, Seoul National University Dental Hospital, 101 Daehak-ro, Jongno-gu, Seoul, 03080 South Korea; 6 Clinical Translational Research Center for Dental Science, Seoul National University Dental Hospital, 101 Daehark-ro, Jongno-gu, Seoul, 03080 South Korea; 7 Dental Life Science Research Institute/Clinical Translational Research Center for Dental Science, Seoul National University Dental Hospital, 101 Daehark-ro, Jongno-gu, Seoul, 03080 South Korea

**Keywords:** Demineralized dentin matrix (DDM), Hepatitis B viruses (HBV), HBV DNA

## Abstract

**Background:**

Demineralized dentin matrix (DDM) is commonly used as a bone-graft substitute. This study measured and compared human hepatitis B viruses (HBV) DNA in fresh dentin to that of dentin processed into DDM extracted during dental treatment from HBV-infected patients. The hypothesis was that the processing procedure for DDM would inactivate or eliminate HBV in the dentin matrix obtained from infected patients.

**Methods:**

Dentin from eighteen HBV-infected patients was collected and each dentin specimen was divided into two fragments. One fragment was used before processing as fresh dentin (control group) and the other was processed into DDM (experimental group). DNA was extracted and purified from each fresh and processed dentin specimen and the HBV DNA copy number quantitated by real time polymerase chain reaction. The HBV DNA copy number in the fresh dentin specimens were compared relative to serologic test results. The second parameter was to evaluate the effectiveness of the processing procedure (defatting, demineralization, freeze-drying, and sterilization) to inactivate or eliminate HBV by comparing the DNA copy number in the processed DDM with that in the matched fresh dentin specimens. All results were analyzed using Mann–Whitney U test to compare numerical measurements between groups and differences were considered statistically significant at P-values less than 0.05.

**Results:**

The presence of HBV DNA was detected in 55.56% (10/18) of the fresh dentin specimens. For the ten HBV DNA-positive fresh dentin specimens, HBV DNA was detected in two (20%) of the matched processed dentin specimens. The copy number of HBV DNA in the two positive processed dentin specimens was 1.79 and 4.03, which were statistically lower than that of the fresh dentin specimens (P = 0.0167).

**Conclusions:**

The results from this study suggested that fresh dentin may be a carrier of HBV and that the procedure used to generate DDM extensively reduced the levels of HBV DNA. Further studies are needed to evaluate the infectivity of HBV in processed dentin.

**Electronic supplementary material:**

The online version of this article (10.1186/s12967-018-1719-9) contains supplementary material, which is available to authorized users.

## Background

Demineralized dentin matrix (DDM) is one of the most acid-insoluble collagenous scaffolds containing minerals and noncollagenous proteins (NCPs) such as bone morphogenetic protein (BMP) and is now commonly used as a bone-graft substitute. Experimental and clinical studies have clearly documented the osteoinductive properties of DDM and its value as an alternative to autogenous bone graft [1, 2]. In addition to the development of autogenous DDM, several in vivo experimental studies on allogenic DDM have been performed and showed promising results for bone repair without any immunologic responses hindering the osteoinductive and osteoconductive capacities of DDM. Subsequently, several clinical studies have been conducted to evaluate the clinical efficacy and safety of allogenic DDM between biological family members [3, 4].

However, in addition to the biologic aspects of allogenic DDM applications, the single largest potential disadvantage might be the risk of transmission of viral disease as has been seen throughout the developmental history of Bone Bank [5, 6]. Even though the biologic use of allogenic DDM has been supported by several experimental and clinical studies, certain viral diseases, such as those caused by human immunodeficiency virus (HIV) and hepatitis B and C viruses may be transmitted through the implantation of human dentin-based products derived from infected donors.

The processing of bone to produce demineralized bone matrix (DBM) entails conditions harsh enough to achieve significant levels of viral inactivation. This allows the pooling process of samples to enhance product quality and effectiveness without increasing, and perhaps even decreasing, the risk of viral transmission [7]. For using DDM as an allogenic bone-graft substitute, the risk of viral-disease transmission should be reduced or eliminated. The processing procedure for producing DDM includes washing, defatting, demineralization, freeze-drying, and sterilization, which removes unwanted materials from the extracted tooth, such as fat, antigens, and inactivated pathogens, while preserving the valuable minerals, collagen matrix, and non-collagenous proteins leading to rapid bone regeneration and bone remodeling [8].

An issue for using DDM as an allogenic bone-graft substitute is whether the allogenic dentin from infected patients could be a carrier for transmissible viral diseases and if there is a potential risk of viral transmission, would the processing procedure reduce or eliminate the risk of transmission. In the current study, hepatitis B virus (HBV) was chosen as the target virus since it is the most prevalent viral infection in the Korean population at 3.7%, is present in saliva, semen, vaginal secretions and serum of infected patients, and since even with careful donor-screening and testing procedures in dental clinics, it is still impossible to be completely sure that an extracted tooth is free of viral contamination [9, 10]. This study was aimed at evaluating viral inactivation by the procedure used to process DDM by measuring HBV DNA in dentin isolated from infected patients both before and after processing. The hypothesis was that dentin obtained from HBV-infected patients could be a carrier of viable HBV to graft recipients through the transplantation and that the processing procedure to generate DDM could completely inactivate or eliminate any HBV present in the dentin matrix.

## Methods

### Tooth collection from HBV-infected patients

This study was approved by the Seoul National University Dental Hospital Institutional Review Board (SNUDH IRB No. CRI17011) and the Seoul National University Bundang Hospital IRB (SNUBH IRB No. B-1705/395-308). All participants signed an informed consent agreement.

Eighteen teeth from individuals chronically infected with HBV were extracted for clinical reasons during dental treatment at SNUDH and SNUBH. The HBV infections were confirmed by serological tests with titers over 1000 international units (IU)/mL (Table 1). All patients were negative for anti-hepatitis C virus (HCV) and anti-HIV antibodies, which allowed for focusing on the hepatitis B virus surface antigen (HBsAg). Vertically infected infants and child patients were excluded. The teeth from the 18 HBV-infected patients were extracted, placed in sterile bottles, and stored in a refrigerator at the dental clinic. The teeth were then aseptically packaged and shipped on dry ice to the Korea Tooth Bank (KTB, Seoul, Korea). Upon arrival at KTB, each tooth was placed in new sterile bottles and stored at − 20 °C until further processing to prevent degradation of the HBV DNA.

**Table 1:** Demographic information and serological status of the patients from whom the teeth used in the study were collected

Patient no.	Sex/age	Tooth number	HBV examination date	Tooth extraction date	HBV examination
					Serology (HBsAg (IU/mL)/anti-HBs
1	F/57	30	02/23/2017	06/05/2017	Positive/< 10.0
2	M/65	30	11/21/2016	07/31/2017	Positive/< 10.0
3	M/54	4	06/26/2017	11/10/2017	Positive/< 10.0
4	M/41	22	07/23/2017	10/13/2017	Positive (5.66)/< 10.0
5	M/37	32	10/16/2017	10/18/2017	Positive (2661.34)/< 10.0
6	F/61	11	10/01/2014	10/30/2017	Positive (2985.25)/< 10.0
7	F/79	17	09/30/2010	10/31/2017	Positive (7305.00)/< 10.0
8	F/63	29	09/16/2011	11/07/2017	Positive (2960.00)/< 10.0
9	F/63	32	09/16/2011	11/07/2017	Positive (2960.00)/< 10.0
10	M/72	12	07/18/2016	11/07/2017	Positive (757.59)/< 10.0
11	F/34	15	11/13/2017	11/13/2017	Positive (5396.97)/< 10.0
12	F/34	17	01/08/2018	01/22/2018	Positive (1447.57)/< 10.0
13	M/29	17	01/05/2018	01/24/2018	Positive (2679.36)/< 10.0
14	M/29	32	01/05/2018	01/24/2018	Positive (2679.36)/< 10.0
15	M/69	30	02/26/2018	03/20/2018	Positive (4784.05)/< 10.0
16	M/35	16	03/05/2018	03/20/2018	Positive (3544.88)/< 10.0
17	F/63	2	01/18/2000	11/28/2017	Positive (4780.80)/< 10.0
18	F/63	31	01/18/2000	11/28/2017	Positive (4780.80)/< 10.0

Eighteen teeth were obtained from patients infected with HBV. All patients were previously diagnosed as being chronically infected with HBsAg titers > 1000 IU/mL. The age range was from 29 to 79 years

HBV, hepatitis B virus; F, female; M, male; HBsAg, hepatitis B surface antigen; anti-HBs, antiviral antibody titer; IU, International Unit

### Experimental design

**Fig. 1:**
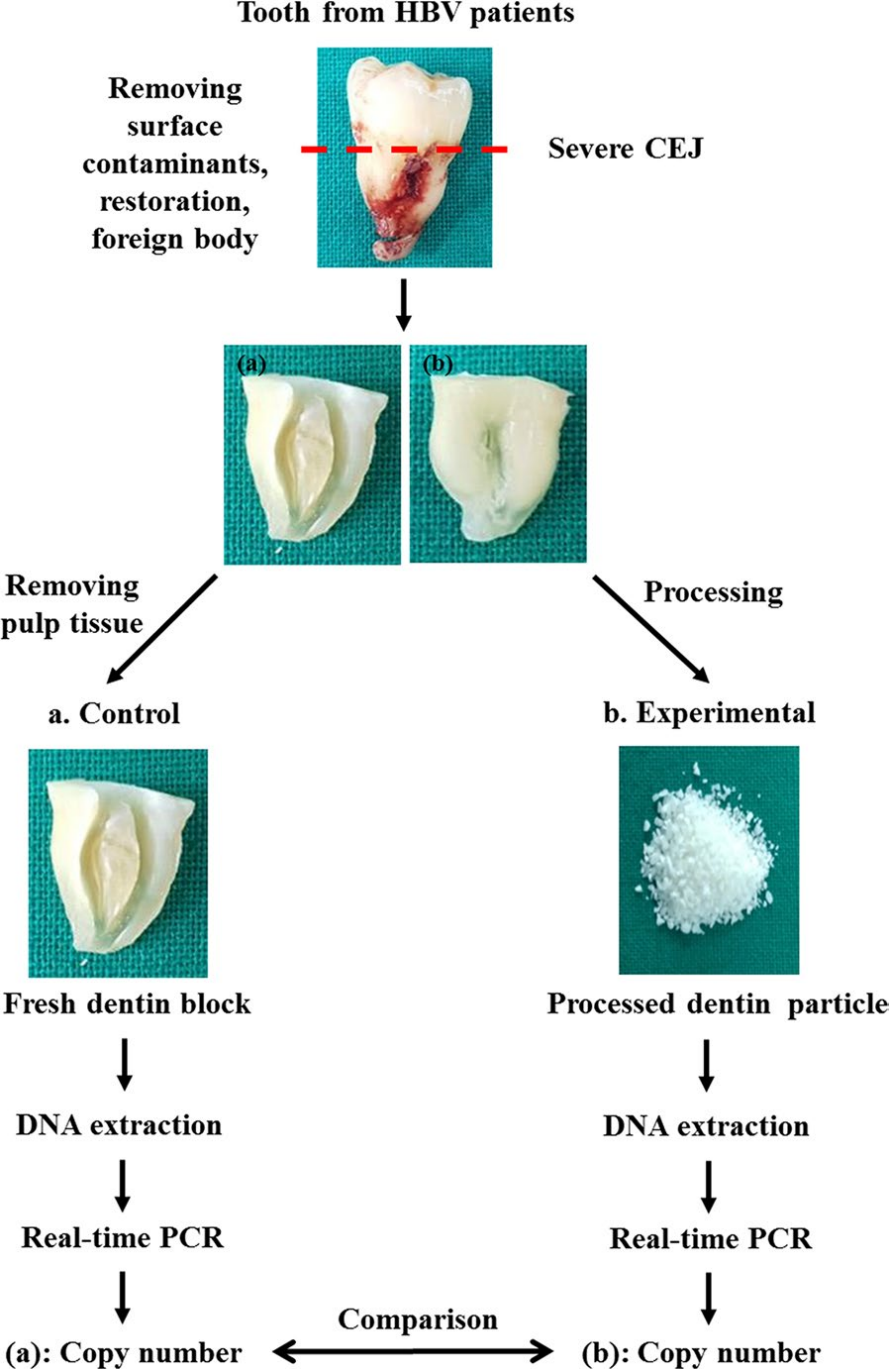
Experimental design flow diagram. Preparation of fresh dentin (control group) and processed dentin (experimental group) for measurement of hepatitis B virus (HBV) DNA. The teeth from chronically-infected HBV patients were severe at the cemento–enamel junction. a A segment of root (control group) cleaned of soft and hard tissues was used as fresh dentin to measure levels of HBV DNA. b Other segment of root (experimental group) was used as processed dentin particles to measure levels of HBV DNA. The HBV DNA of fresh dentin (control group) measured as copy number by quantitative polymerase chain reaction was compared that of processed dentin (experimental group)

The dentin obtained from the HBV-infected patients was divided into two pieces. One piece was used for HBV DNA measurement in fresh dentin (control group) and the second piece was processed for production of DDM (processed dentin) and then analyzed for HBV DNA. The comparison of HBV DNA levels in the fresh dentin compared with that in the processed dentin was intended to evaluate the effectiveness of the processing procedure to inactivate or eliminate HBV in dentin (Fig. 1).

### Preparation of fresh dentin and processed dentin

Each tooth was cleaned of all soft tissues, cavities, calculus, restoration, and root canal materials using a dental high-speed bur (FG 6856 018 Diamond Bur; Gebr. Brasseler GmbH & Co, Lemgo, Germany) and washed by saline irrigation. The enamel portion over the cemento-enamel-junctions was severed using a FG700 010 Cutting Bur. Root cementum was removed by scraping the surface using #15 surgical blade and FG 6856018 Diamond Bur. The remaining root portion was divided into two pieces using a dental cutting bur (FG700 010 Cutting Bur; Gebr. Brasseler GmbH & Co). All dental pulp, soft tissues, and attached hard tissues on the internal and external surfaces were cleaned using a dental handpiece.

The pieces used as fresh dentin for measuring the level HBV DNA were washed three times with sterilized water and then dried in a chamber at 56 °C for 2 h (Fig. 1a). The other pieces were processed to DDM particles as autogenous tooth bone graft material (AutoBT) according to the previous report by the Korea Tooth Bank [8]. Briefly, the dentin segments were ground and sieved to yield particles of 300 to 800 μm in size, washed, defatted, demineralized, freeze dried, and sterilized, all under temperature-controlled sonication (40 kHz) and stirring. The particles were soaked in hydrogen peroxide (H_2_O_2_) solution for 30 min and then washed repeatedly with distilled water. The particles were then immersed in 70% ethyl alcohol for 1 h. The defatted powders were washed three times with sterile distilled water for 10 min to remove the chemicals used in the processing. The particulate dentin was demineralized to 10 to 30% residual calcium (by weight) using 0.6 N HCl solution at room temperature for 3 min. The demineralized dentin particles were then washed three times for 10 min with sterile distilled water and a sodium phosphate buffer to remove residual acid. Next, the demineralized dentin particles were soaked in 70% ethanol and H_2_O_2_ for 30 min at room temperature and then rinsed with sterile distilled water. The particles were freeze-dried over night to less than 5% residual moisture (by volume). The particles were packaged and finally sterilized with ethylene oxide gas (Fig. 1b).

## HBV DNA measurements

### DNA isolation and purification

In preparation for analysis by quantitative polymerase chain reaction, individual samples of fresh and processed dentin (0.5 g) were decalcified and digested using a Tbone EX Kit (DNA Chip Research Inc., Tokyo, Japan) followed by phenol–chloroform extraction. Total DNA was extracted according to the manufacturer’s instructions. Briefly, the dentin samples were placed in 50-mL tubes containing 30 mL Solution A and soaked at 23 °C for 12 h. We then added 1.8 mL Solution B to each tube containing Solution A and the sample. The mixture was gently agitated at 37 °C for 2 h and then centrifuged (13,000 rpm, 5 min). Following this, the supernatant was discarded and 400 µL of Solution C and 50 µL Proteinase K (20 mg/mL) were added to the sample. The mixture was incubated at 56 °C with gentle agitation for 3 h (Additional file 1).

The DNA solution from each sample was purified using a QIAamp DNA Investigator Kit (Qiagen, Hilden, Germany). The DNA samples were then purified using a silica membrane following the manufacturer’s instructions and as detailed in the Additional file 1. The purified DNA was eluted in 100 µL of Buffer ATE (Additional file 1).

### Quantitative polymerase chain reaction

Quantitative real-time PCR (qPCR) was performed using a 7900HT Fast Real-Time PCR System (Applied Biosystems, Foster city, CA, USA) and a Hepatitis B Virus PCR Kit (MyBiosource, Inc., San Diego, CA, USA) with oasig lyophilized 2× qPCR Mastermix (Primerdesign, Ltd., Camberley, UK). The PCR reactions were set up according to the manufacturer’s instructions by aliquoting 15 μL of master mix into each reaction well of a 96-well reaction plate (Applied Biosystems, Foster city, CA, USA) followed by 5 μL of each experimental DNA sample for a final volume of 20 μL per reaction. Primer details are included in Additional file 1. Six concentrations of a reference DNA sample (the ‘Standard DNA’) were prepared by serial dilution and analyzed in triplicate for each 96-well plate in the study. All experimental DNA samples were assayed in triplicate. The thermal cycling profile included Stage 1: 2 min at 95 °C; Stage 2: 50 cycles of 10 s at 95 °C and 60 s at 60 °C with signal acquisition (Additional file 1).

### Data and statistical analysis

The data were analyzed with the real-time PCR thermal cycler software (SDS 2.4.1 for the 7900HT). Fluorescence data were normalized to the ROX signal and the baseline signal and threshold were set automatically. The HBV DNA copy number in the fresh dentin was compared with that in the processed dentin of same tooth. Cut off levels and specificity were not determined due to the limited number of human dentin samples.

All statistical analysis was performed using R Statistical Software (version 3.5.1; R Foundation for Statistical Computing, Vienna, Austria). Mann–Whitney U test was used to compare numerical measurements between groups and differences were considered statistically significant for P-values were less than 0.05.

## Results

### HBV DNA in fresh dentin

**Fig. 2:**
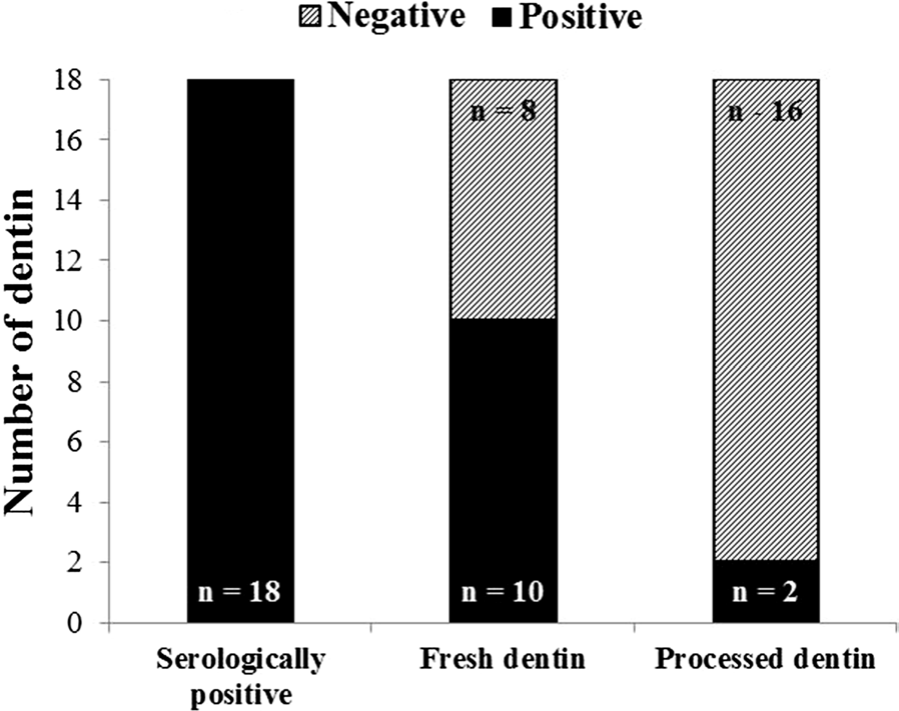
Number of dentin samples from serologically hepatitis B virus (HBV)-positive patients, the number of HBV DNA-positive fresh dentin samples, and the number of HBV DNA-positive processed dentin samples. Among eighteen dentins from HBV-infected patients, ten fresh dentin samples (55.6%) contained detectable HBV DNA. HBV DNA of the other eight fresh dentins (44.4%) was considered degraded or insufficient levels of DNA available for detection. No significant difference was observed between the serologically positive dentin and HBV DNA-positive fresh dentin (P = 0.3536) based on linear regression analysis (univariate) of the relationship. HBV DNA from eight of the ten fresh dentin samples (80%) that were positive for HBV DNA appeared degraded or eliminated by the processing. Two (patients #3 and #14) of the ten fresh dentin samples that were positive HBV DNA (20%) were extensively degraded after processing

Among the eighteen dentins obtained from HBV-infected patients, ten fresh dentin specimens (55.6%) contained HBV DNA. HBV DNA in the other eight fresh dentins (44.4%) was not detected owing to insufficient DNA amounts or quality needed for analysis (Fig. 2). Linear regression analysis (univariate) of the correlation between HBV copy number in the fresh dentin and the HBsAg positivity based on patient serology was not statistically significant (P = 0.3536).

### HBV DNA in processed dentin

HBV DNA in eight of the ten fresh dentin samples (80%) that were positive for HBV DNA appeared to be completely degraded or eliminated by the DDM processing. Samples from two of the ten patients (20%) whose fresh dentin were positive for HBV DNA were extensively degraded after processing (#3 and #14). The difference between the fresh dentin and processed dentin with respect to HBV DNA-positivity was confirmed by multivariable logistic regression analysis with a *P* value = 0.0167 confirming the statistical significance.

**Fig. 3:**
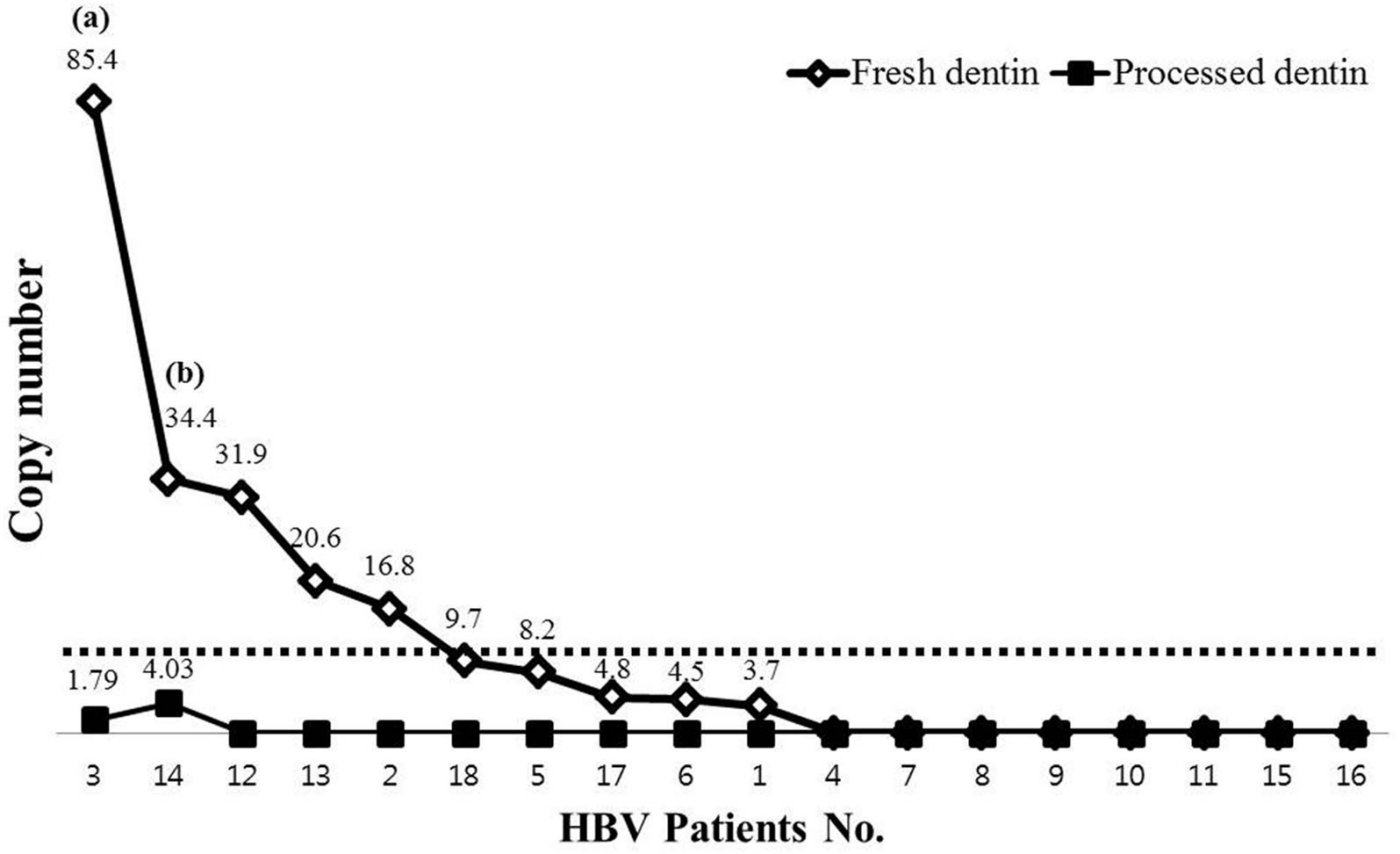
Change in copy number of hepatitis B virus (HBV) DNA from fresh dentin to processed dentin. Copy numbers of 1.79 and 4.03 in two processed dentin samples (patients 3 and 14) corresponded with the fresh dentin samples containing the highest (**a**) and second highest (**b**) copy numbers (85.42 and 34.4, respectively) of HBV DNA were considered extensively degraded. Eight fresh dentin samples with HBV DNA copy numbers less than 31.9 were negative for HBV DNA in the processed dentin and considered to be completely degraded (inactivation or elimination of HBV). A copy number less than 10 is generally considered negative for virus, even though the cut off levels were not determined in the current experiment. With P < 0.05 being considered statistically significant, the correlation of copy number between fresh dentin and processed dentin was confirmed by multivariable logistic regression and determined to be statically significant (P = 0.0167)

HBV DNA copy numbers of 1.79 and 4.03 in the processed dentin of patients 3 and 14, respectively, corresponded with the highest and second highest copy numbers (85.42 and 34.4, respectively) in the fresh dentin samples, which demonstrated that the samples underwent extensive degradation during the DDM processing. The eight fresh dentin samples with HBV DNA copy numbers less than 31.9 were all negative in their corresponding processed dentin samples and were considered as complete inactivation or elimination of HBV (Fig. 3, Additional file 2).

## Discussion

The purpose of the current study was to evaluate the ability of a processing procedure for the generation of DDM to inactivate or eliminate HBV in dentin obtained from chronically infected patients by comparing the HBV DNA levels in processed dentin with those in fresh dentin. It was our hypothesis that the processing procedure would degrade nucleic acids, inactivating HBV and rendering it noninfectious. The viral burden in fresh dentin might be a primary factor for determining the processing method to prevent HBV transmission since the removal of HBV DNA in processed dentin might assure the safety for the allogenic application of dentin. Validation of viral inactivation by the processing procedure could lead to the expanded application of allogenic DDM in the dental field.

Based on the results of the current study, dentin may be a potential carrier of HBV since 55.6% (10/18) of the fresh dentin samples that were stored refrigerated without removal of blood, saliva, or other foreign bodies at the dental clinic tested positive for HBV DNA by qPCR.

Eight of the processed dentin samples among the ten HBV DNA-positive fresh dentin samples (80%) showed complete degradation of nucleic acid of HBV DNA with the reduction being statistically significant. HBV DNA persisted in two of the processed dentin samples among the ten HBV DNA-positive fresh dentin samples (20%). These two corresponded with the fresh dentin samples containing the highest and second highest copy number of HBV DNA 85.42 and 34.4 compared to 1.79 and 4.03, respectively, post processing. A copy number less than 10 is generally considered to be negative for virus even though the actual cut off levels and specificity were not determined in the current experiment.

There have been several approaches used beyond standard donor testing and screening procedures in the developmental history of bone banks to prevent viral transmission. The use of H_2_O_2_ as an oxidizing chemical is used to process bone allografts in effort to eradicate microorganisms and viruses. Viral clearance following a 1 h H_2_O_2_ treatment verifies that the risk for disease transmission can be greatly reduced or eliminated by greater than six logs sterility assurance level (SAL), except for the porcine parvovirus (PPV) virus [11]. SAL is the probability that an item will not be sterile after it has been subjected to a validated sterilization process. With a SAL of 10^−6^, the odds of an organisms surviving after allograft processing are less than one in one million [5]. Processed bone allografts obtained from an HIV-infected donor and cleaned with a 30% ethanol solution and rinsed in 100% ethanol prior to lyophilization failed to transmit the virus to the graft recipient, whereas unprocessed bone allografts obtained from the same donor transmitted the virus [12]. Proprietary solutions, including those used for chemical sterilization with ethylene oxide, may contain particular bactericidal, viricidal, and fungicidal agents but there is no industry-wide standard for their use [13]. Freeze-drying is a process by which water is removed from the tissue to the point where cellular activity is no longer supported, which may inactivate HIV and HCV and reduce the risk of transmission by infected blood products and bone marrow [14].

Although the processing of dentin employs steps of washing, defatting, demineralization, freeze-drying, and sterilization with ethylene oxide gas, the majority of studies on viral clearance has been focused on the demineralization process in relation to the development of demineralized freeze-dried bone allograft (DFDBA). In the processing of preparing DFDBA, investigators have demonstrated that exposing allografts to low-pH solutions such as hydrochloric acid inactivates numerous viruses, including HIV, HBV, HCV, cytomegalovirus, and poliovirus [15–17]. Scarborough et al. performed a study to validate the effectiveness of a bone demineralization process with respect to its inactivation of viruses, including HIV, duck hepatitis B virus (a model for human hepatitis B), bovine viral diarrheal virus (a model for human hepatitis C), human cytomegalovirus, and human poliovirus (a model for small non-enveloped viruses such as hepatitis A). The infectivity of all RNA and DNA viruses is reduced more than one-million-fold (10^−6^) for all the viruses tested and as much as one-trillion-fold (10^−12^) for poliovirus. For example, the probability of HIV survival after bone demineralization is less than 1 in 2.8 billion [15, 17]. The demineralization method degrades nucleic acids in retrovirus-infected cortical bone and thereby preventing disease transmission through the allotransplantation of DBM powder. The ability of a demineralization procedure to effectively inactivate an infectious retrovirus in systemically infected bone while maintaining the desired osteoinductive properties of powdered DBM appears to provide an additional margin of safety while sustaining optimal allograft efficacy [18].

The Centers for Disease Control and Prevention (CDC) report that DFDBA materials are widely used in periodontal and dental therapy and that there are no reports of disease transmission during the 30-year history of using freeze-dried bone allografts [19]. There has also been no report of disease transmission (HIV or hepatitis viruses) using demineralized bone products (FDBA, DFDBA, DBM) [20].

DDM consists of osteoconductive type 1 collagen and noncollagenous proteins, including osteoinductive bone morphogenetic proteins (BMPs), which stimulate the formation of bone at a defect site similar to that treated with DBM that is washed, demineralized with organic solvents, freeze-dried, and sterilized, resulting in a significant level of viral inactivation as previously mentioned [1, 21]. The major differences between DDM and DBM are that bone contains viable osteocytes and blood vessels (harversian canals and endothelial cells) while there are no cells or blood vessels in dentin that could be a potential source of transmissible viruses [22]. Tissues obtained from living donors have lower rates of bacterial contamination than tissues harvested from cadavers at autopsy [23]. Likewise, because dentins are obtained from patients during dental treatment, the possibility of disease transmission from dentin might be lower than that from a bone allograft. For the described reasons, a dentin allograft might be safer than a bone allograft.

However, while we measured HBV DNA levels in processed dentin, the precise step of washing, defatting, demineralization, or freeze-drying that resulted in the degradation of the virus, either alone or in combination, was not determined. Therefore, more studies to determine the effects of the various steps of dentin processing on the degradation of HBV and other viruses are warranted.

Of note, we currently do not know whether or not the positive HBV DNA in processed dentin was infectious since we did not determine the cutoff levels of HBV DNA in this experiment. Since qPCR techniques may amplify a small segment of degraded HBV DNA in the absence of intact virus, additional studies are necessary to determine whether the detected HBV DNA that remained in the processed dentin samples correlates with infectious virus. Finally, the established validation of viral inactivation procedures, including the harvesting of tissue in a sterile manner, repeated washings, immersion in ethanol, freeze-drying, demineralization, and sterilization, should be confirmed for rendering a safe DDM allograft [24, 25].

## Conclusions

The results of the current study suggested that the positive detection of HBV DNA in 10 (55.6%) fresh dentin samples may be indicative of infected dentin, which may serve as potential carriers of HBV. A processing procedure for producing DDM completely degraded the HBV DNA in eight of the ten fresh dentin that were positive for HBV DNA. This reduction was statistically significant. The two processed dentin samples with extensively degraded HBV DNA that remained positive need to be evaluated for infectivity. For the purpose of achieving completely virus-free grafting specimens, additional studies are necessary, including those to validate viral inactivation to determine whether or not the processed dentin samples that were positive for HBV DNA were infectious. The ability of the processing procedure for DDM to effectively inactivate HBV in specimens from infected patients appears to provide an expanded margin of safety for the clinical application of allogenic DDM from the bench to chairside.

## Additional files

**Additional file 1.** Key resources in HBV DNA measurement.

**Additional file 2.** Hepatitis B Virus copy numbers in fresh and processed dentin from infected patients.

## Abbreviations

BMP: bone morphogenetic protein
CDC: center for disease control and prevention
DBM: demineralized bone matrix
DDM: demineralized dentin matrix
DFDBA: demineralized freeze-dried bone allograft
HBV: human hepatitis B viruses
HIV: human immunodeficiency virus
IU: international unit
NCPs: noncollagenous proteins
SAL: sterility assurance level
