# Supplementary material for: Measurement of hepatitis B virus DNA in fresh versus processed dentin from chronically infected patients
Source: J Transl Med. 2018 Dec 12;16:351. doi: 10.1186/s12967-018-1719-9 (PMC6292124; doi:10.1186/s12967-018-1719-9)
Supplement: Supplementary file 1 — Additional file 1. Key resources in HBV DNA measurement. [file 12967_2018_1719_MOESM1_ESM.pdf]

Additional files

Additional file 1. Key resources in HBV DNA measurement

Reagent or Resource	Source	Identifier
**Reagents**		
Proteinase K	Ambion	Cat#AM2546
Phenol:Chloroform:Isoamyl Alcohol 25:24:1, Saturated with 10mM Tris, pH 8.0, 1mM EDTA	Sigma	Cat#P3803
**Critical commercial assays**		
TBONE EX Kit	DNA Chip Research Inc.	Cat#TBEW200
QIAamp DNA Investigator Kit	Qiagen	Cat#56504
Lyophilsed 2X qPCR MasterMix	Primerdesign Ltd.	Cat#oasig-standard-150
Real-time PCR detection kit for Hepatitis B Virus	MyBiosource Inc.	Cat#MBS486092
**Software**		
SDS 2.4.1	Applied Biosystems	https://www.thermofisher.com/kr/ko/home/technical-resources/software-downloads/applied-biosystems-7900ht-fast-real-timespcr-system.html
