# Supplementary material for: Measurement of hepatitis B virus DNA in fresh versus processed dentin from chronically infected patients
Source: J Transl Med. 2018 Dec 12;16:351. doi: 10.1186/s12967-018-1719-9 (PMC6292124; doi:10.1186/s12967-018-1719-9)
Supplement: Supplementary file 2 — Additional file 2. Hepatitis B Virus copy numbers in fresh and processed dentin from infected patients. [file 12967_2018_1719_MOESM2_ESM.pdf]

Additional files

Additional file 2. Hepatitis B Virus copy numbers in fresh and processed dentin from infected patients

	**Patient No.**	**3**	**14**	**12**	**13**	**2**	**18**	**5**	**17**	**6**	**1**	**4**	**7**	**8**	**9**	**10**	**11**	**15**	**16**
**Copy number**	**Fresh dentin**	85.4	34.4	31.9	20.6	16.8	9.7	8.2	4.8	4.5	3.7	0	0	0	0	0	0	0	0
	**Processed dentin**	1.79	4.03	0	0	0	0	0	0	0	0	0	0	0	0	0	0	0	0

Copy number is 3.2Kb HBV genomic DNA. Copy number is in descending levels of fresh dentin.

Copy number of 1.79 and 4.03 in two processed dentin (patients 3 and 14) were from first (a) and second (b) high copy numbers (85.42 and 34.4 each) in fresh dentin that considered extensively degraded. Eight fresh dentin with copy number below 31.9 were all changed to copy number 0 in processed dentin that considered completely degraded (inactivation or elimination of HBV). The correlation of positive HBV DNA between 10 fresh dentin ad 2 processed dentin is statistically significant ($p=0.0167$) at $P< 0.05$. The copy number below 10 is generally considered as there is no virus even though the cut off levels were not determined in this experiment.
